# Evaluation of direct costs associated with alveolar and cystic echinococcosis in Austria

**DOI:** 10.1371/journal.pntd.0007110

**Published:** 2019-01-31

**Authors:** Felix Lötsch, Christine M. Budke, Herbert Auer, Klaus Kaczirek, Fredrik Waneck, Heimo Lagler, Michael Ramharter

**Affiliations:** 1 Division of Infectious Diseases and Tropical Medicine, Department of Medicine 1, Medical University of Vienna, Vienna, Austria; 2 Institut für Tropenmedizin, Universität Tübingen, Tübingen, Germany; 3 Department of Veterinary Integrative Biosciences, College of Veterinary Medicine & Biomedical Sciences, Texas A&M University, College Station, Texas, United States of America; 4 Department of Medical Parasitology, Institute of Specific Prophylaxis and Tropical Medicine, Center of Pathophysiology, Infectiology and Immunology, Medical University of Vienna, Vienna, Austria; 5 Department of Surgery, Medical University of Vienna, Vienna, Austria; 6 Division of Cardiovascular and Interventional Radiology, Department of Biomedical Imaging and Image-guided Therapy, Medical University of Vienna, Vienna, Austria; 7 Department of Tropical Medicine, Bernhard Nocht Institute for Tropical Medicine & I. Department of Medicine, University Medical Center Hamburg-Eppendorf, Hamburg, Germany; IRNASA, CSIC, SPAIN

## Abstract

**Background:**

Cystic echinococcosis (CE) is a globally occurring zoonosis, whereas alveolar echinococcosis (AE) is endemic only in certain parts of the Northern Hemisphere. The socioeconomic impact of human echinococcosis has been shown to be considerable in highly endemic regions. However, detailed data on direct healthcare-related costs associated with CE and AE are scarce for high income countries. The aim of this study was to evaluate direct costs of human disease caused by CE and AE in Austria.

**Methods:**

Clinical data from a registry maintained at a national reference center for echinococcosis at the Medical University of Vienna were obtained for the years 2012–2014. These data were used in conjunction with epidemiological data from Austria’s national disease reporting system and diagnostic reference laboratory for echinococcosis to assess nationwide costs attributable to CE and AE.

**Results:**

In Austria, total modelled direct costs were 486,598€ (95%CI 341,825€ – 631,372€) per year for CE, and 683,824€ (95%CI 469,161€ - 898,486€) for AE. Median costs per patient with AE from diagnosis until the end of a 10-year follow-up period were 30,832€ (25^th^– 75^th^ percentile: 23,197€ - 31,220€) and 62,777€ (25^th^– 75^th^ percentile: 60,806€ - 67,867€) for inoperable and operable patients, respectively. Median costs per patients with CE from diagnosis until end of follow-up after 10 years were 16,253€ (25^th^– 75^th^ percentile: 8,555€ - 24,832€) and 1,786€ (25^th^– 75^th^ percentile: 736€ - 2,146€) for patients with active and inactive cyst stages, respectively. The first year after inclusion was the most cost-intense year in the observed period, with hospitalizations and albendazole therapy the main contributors to direct costs.

**Conclusions:**

This study provides detailed information on direct healthcare-related costs associated with CE and AE in Austria, which may reflect trends for other high-income countries. Surgery and albendazole therapy, due to surprisingly high drug prices, were identified as important cost-drivers. These data will be important for cost-effectiveness analyses of possible prevention programs.

## Author summary

Cystic and alveolar echinococcosis, caused by *E*. *granulosus* and *E*. *multilocularis*, both occur in humans in Austria. Lesions may develop at any site, with the liver being most frequently affected. Morbidity–especially for alveolar echinococcosis–can be significant and treatment may include major surgery or long-term suppressive medical therapy. The present study was performed to investigate direct healthcare-related costs of cystic and alveolar echinococcosis in Austria based on the data from a clinical registry maintained at the reference center for echinococcosis, the reference laboratory for echinococcosis and data from the national disease reporting system. Annual incidences of AE and CE were estimated at λ = 12 and λ = 40 newly diagnosed cases per year, respectively. Estimated costs due to cystic echinococcosis were 486,598€ (95%CI 341,825€ – 631,372€) per year, and 683,824€ (95%CI 469,161€ - 898,486€) for AE. Major cost drivers were surgical interventions with hospitalizations and drug costs. These data will provide a basis for cost-effectiveness analyses of prevention programs, and highlight possible targets for cost reduction strategies.

## Introduction

Cystic echinococcosis (CE) is a zoonosis caused by *Echinococcous granulosus* and is endemic on all continents. In contrast, *Echinocococcus multilocularis*, the pathogen responsible for alveolar echincoccosis (AE), occurs only in certain areas of the Northern Hemisphere [1]. Both infections occur in humans in Austria. The western provinces of Austria are traditional hotspots of *E*. *multilocularis* transmission, with an increase in cases seen recently [2]. The geographic distribution of AE has expanded over the past several decades and now includes the entire country. Autochthonous *E*. *granulosus* transmission is rare in Austria, with only occasional locally-acquired CE cases reported in the past 20 years [3]. The majority of CE cases treated in Austrian hospitals are migrants from South-Eastern Europe and the Middle East, residing foremost in Austria’s Eastern provinces.

AE is associated with significant morbidity and mortality if left untreated. Treatment varies with disease stage, but therapeutic options are typically limited to hepatic surgery and albendazole therapy. If surgery is not feasible, palliative management with long-term albendazole treatment is indicated. Ideally, AE cases should receive long-term follow-up with advanced radiological imaging modalities, including positron-emission-tomography/computed tomography (PET/CT) [4]. CE is treated using a cyst stage-specific scheme consisting of surgery, percutaneous interventions, pharmacological treatment, or a watch-and-wait approach [5][6].

Although the socioeconomic impact of echinococcosis has previously been assessed for endemic countries [7], detailed studies on direct costs linked to echinococcosis, and in particular AE, are scarce and even more so for high-income regions.

Consequently, there is a dearth of information about the economic impact of AE and CE in high income regions like the Central European country of Austria, which may guide decision makers about the cost-effectiveness of control measures and of clinical management. The aim of this study was, therefore, to quantify costs associated with the treatment of echinococcosis in Austria from a societal perspective.

## Methods

Clinical and epidemiological data were collected from a clinical registry of CE and AE patients managed at a reference center for the clinical management of echinococcosis at the General Hospital (AKH) of the Medical University of Vienna in Austria from 2012 to 2014. Patients seeking care for the first time in the given time period and subsequently managed at the reference center were eligible to be included. However, not all echinococcosis patients are registered, referred or managed at this center with other large centers treating patients on an individual basis. Thus, additional epidemiological data on the incidence of echinococcosis in Austria were collected from the Austrian Ministry of Health’s report on zoonoses [8] and the national reference laboratory for the diagnosis of echinococcosis, which is part of the Department of Parasitology at the Medical University of Vienna, and the only reference laboratory for echinococcosis in Austria. This laboratory processes samples from all suspected cases of echinococcosis in Austria. Ethics approval was obtained from the ethics committee of the Medical University of Vienna (#2031/2012).

Frequency data on hospitalizations and interventions were extracted from the clinical registry and patient records. Costs associated with these hospitalizations were obtained from the hospital’s Office for Medical Economics [5]. Data were also collected from patient records on the duration of albendazole drug therapy, including deviations from standard dosing. When complete dosing information was not available, these data were imputed based on published guidelines [5][6]. For outpatient routine laboratory testing (complete blood count and standard chemical parameters), specific diagnostic tests for echinococcosis (ELISAs, Western Blots, PCRs and histological examinations), clinical follow-up visits, diagnostic imaging (CT-scans, magnetic resonance imaging (MRI) scans, PET/CT and PET/MRI), and albendazole treatment, cost estimates were obtained from the Department of Parasitology, the hospital’s outpatient self-payer guide (not freely available), and the Austrian Reimbursement Code (“Erstattungskodex”) for registered drugs [9].

Overall direct costs for incident cases presenting to the AKH in the years 2012, 2013, and 2014 were summed and the proportional contribution of respective items (hospitalizations, imaging, etc.) analyzed per year of follow-up. Patients were classified as suffering from AE or CE, and according to CE-specific disease stage at inclusion into the study. Cost data were assessed for normality and the Wilcoxon matched-pairs signed-ranks test used to compare costs for the year of diagnosis and subsequent two years of follow-up. In order to model nationwide costs, estimates of incidence were based on data from Austria’s diagnostic reference laboratory for echinococcosis in addition to national data provided by the Ministry of Health. Data from these institutions represent the best available estimates of AE and CE incidence in Austria.

A spreadsheet model was developed in Microsoft Excel 2010 for Windows to evaluate national direct costs associated with AE and CE. Parameters were sampled across their distributions using a Monte Carlo approach with 1,000 bootstrap replicates. Annual incidence was modelled using a Poisson distribution with λ = 12 for AE and λ = 40 for CE [2]. Mean patient age was estimated at 60 years for AE and 44 years for CE based on clinical registry data. A year 2013 Austrian life table was used to model yearly survival probability for patients aged 60 and 44 years, respectively [10]. The proportion of patients receiving an intervention (surgery or interventional radiology) was modeled using a binomial distribution based on clinical registry data, with probability = 0.65 for AE and probability = 0.66 for CE. The seemingly comparable proportion of patients requiring an intervention for both AE and CE is explained by the fact that minimally invasive procedures such as PAIR (puncture, aspiration, injection, reaspiration) are part of the estimate for CE. A follow-up time of 10 years was assumed according to the local standard operating procedure. Direct costs for the first 3 years of treatment were obtained based on registry data. For year 4 through 10, yearly costs were estimated to be 90% of the previous year until the end of follow-up, for both AE and CE. In a previous Swiss study on AE [11], a discount factor of 3% was applied. However, this factor is believed to overestimate costs for the population under care in Austria. Therefore, in order to account for monitoring and the long-term use of albendazole, the current study includes a slightly steeper decrease in costs over time. In order to evaluate the impact of this discounting factor, a sensitivity analysis was performed by comparing the results to a model without discounting (i.e., assuming year 3 costs would be applicable for years 4–10) and to a model with 3% discounting per year.

## Results

In total, 45 patients (7 patients with AE and 38 with CE) presented for the first time to the center between 2012 and 2014. Of the 38 patients with CE, 8 were first diagnosed in 2012, 17 were first diagnosed in 2013, and 13 were first diagnosed in 2014. Type of infection, anatomical location of cysts, and CE hepatic cyst stage are shown in Table 1. Of the evaluated patients with CE, 16/38 (42%) were male and the mean age was 44 years (median 45 years, range 8 to 81 years) at inclusion. Out of the 7 AE patients, 2 were first diagnosed in 2012, 0 were first diagnosed in 2013, and 5 were first diagnosed in 2014. One (14%) AE patient was male and mean patient age was 60 years (median 62 years, range 27 to 81 years) at inclusion. No deaths occurred in the observed period.

**Table 1:** Number of patients with AE and CE according to cyst stage.

**Stage**	**Number of patients (n = 45)**
**AE (all hepatic)**	7
**CE** **Hepatic** ***CE1*** ***CE2*** ***CE3a*** ***CE3b*** ***CE4*** ***CE5*** **Pulmonary** **Musculo-skeletal** **Pancreas** **Genitourinary** **Peritoneum**	38* 31 *5* *4* *7* *9* *3* *3* 6 4 1 2 1

*7 patients had cysts in more than one location.

### Cystic echinococcosis

**Fig 1:**
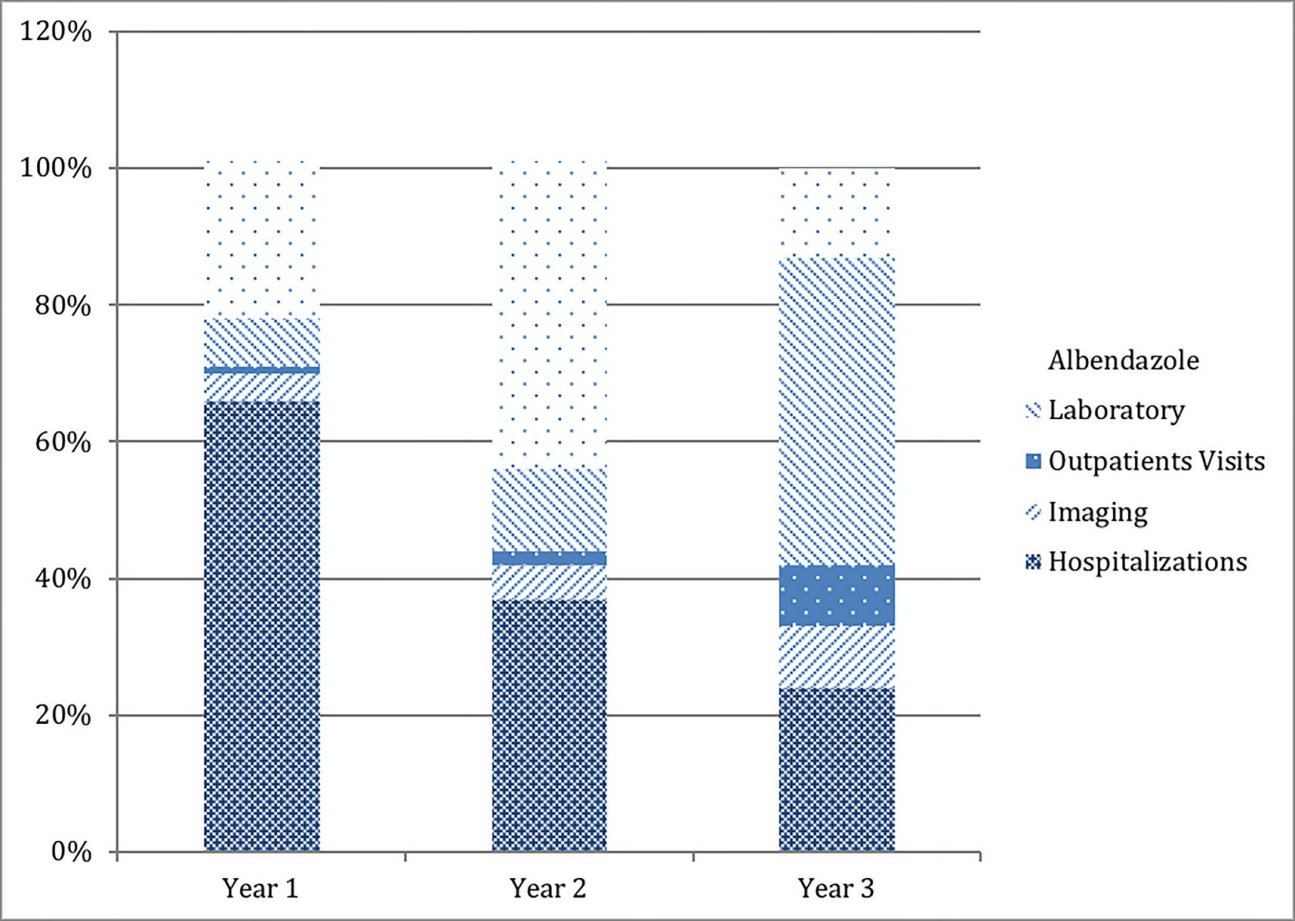
Proportional contribution of each cost item to direct, CE-associated costs for years one, two, and three of follow-up.

Based on time of follow-up, overall direct, healthcare-related cost associated with CE for the first year after diagnosis at our center was 336,419€ (n = 38 patients), 70,619€ for the second year (n = 25) and 5,335€ (n = 8) for the third year (see Table 2). The most cost-intensive components in the first year of treatment were hospitalizations (66%), followed by albendazole therapy (23%), whereas laboratory procedures (7%), diagnostic imaging (4%), and outpatient visits (1%) only contributed to a small proportion of direct healthcare-related costs. Proportional costs per year of follow-up are depicted in Fig 1. Per patient costs were highest in the year of diagnosis (8,853€) and significantly decreased in the second year of treatment (2,824€) (p = <0.001; n = 25). Cost per patient continued to decline into the third year of treatment (667€). However, there was not a statistically significant difference between year 2 and year 3 (p = 0.161; n = 8). For year 1, a stage-based analysis showed that per-patient costs were higher for active cysts (CE1 to CE3b and extrahepatic active cysts combined) compared to inactive cysts (CE4 and CE5 combined) (see Table 3) (p = 0.001).

**Table 2:** Item-specific costs associated with the treatment of CE cases (n = 38) at an Austrian reference center for echinococcosis for the first 3 years of treatment. As total costs (last row) are based on a decreasing number of patients each year, this value should only be interpreted as a reference value for the proportion of costs each item is contributing.

Items	Year 1 (% of overall costs); n = 38 patients	Year 2 (% of overall costs); n = 25 patients	Year 3 (% of overall costs); n = 8 patients
**Interventions including hospitalizations**	**220,640€ (66%)**	**25,955€ (37%)**	**1,281€ (24%)**
Hepatic surgery	143,454€ (n = 13)	0€ (n = 0)	0€ (n = 0)
Percutaneous treatments	13,416€ (n = 8)	1,281€ (n = 1)	1,281€ (n = 1)
Non-hepatic surgery	49,412€ (n = 6)	16,684€ (n = 2)	0€ (n = 0)
Other CE-related hospitalizations; diagnostic or management of complications	14,358€ (n = 5)	7,990€ (n = 1)	0€ (n = 0)
**Imaging**	**13,265€ (4%)**	**3,292€ (5%)**	**455€ (9%)**
Magnetic Resonance Imaging	5,814€ (n = 34)	1,710€ (n = 10)	342€ (n = 2)
Computed Tomography	6,780€ (n = 60)	1,582€ (n = 14)	113€ (n = 1)
PET-CT	671€ (n = 1)	0€ (n = 0)	0€ (n = 0)
**Outpatient visits**	**4,149€ (1%) (n = 142)**	**1,549€ (2%) (n = 53)**	**497€ (9%) (n = 17)**
**Laboratory**	**22,225€ (7%)**	**8,225€ (12%)**	**2,403€ (45%)**
Standard laboratory packages	14,410€ (n = 55)	6,288€ (n = 24)	1,834€ (n = 7)
Serology	6,036€ (n = 147)	1,858€ (n = 47)	569€ (n = 12)
Histology	603€ (n = 15)	0€ (n = 0)	0€ (n = 0)
PCR	1,177€ (n = 15)	78€ (n = 1)	0 €(n = 0)
**Albendazole (n = packs)**	**76,140€ (23%) (n = 653)**	**31,599€ (45%) (n = 271)**	**700€ (13%) (n = 6)**
**Total**	**336,419€**	**70,619€**	**5,335€**

**Table 3:** Average per-patient costs for active and inactive (CE4 and CE5) CE cyst stages per year of follow-up.

**Stage**	**Average cost per patient in year 1 (n = 38)**	**Average cost per patient in year 2 (n = 25)**	**Average cost per patient in year 3** **(n = 8)**
**Active stages**	10,230€	3,002€	758€
**Inactive stages (CE4 & CE5)**	1,705€	785€	29€
**All stages**	8,853€	2,824€	667€

Assuming 10% discounting in years 4–10, modelled median costs per patients with CE from diagnosis until end of follow-up after 10 years were 16,253€ (25^th^– 75^th^ percentile: 8,555€ - 24,832€) and 1,786€ (25^th^– 75^th^ percentile: 736€ - 2,146€) for patients with active and inactive cyst stages, respectively. Based on an estimated incidence of 40 cases per year, a mean age of 44 years, and a 66% probability of requiring an invasive intervention (surgery or interventional radiology), the modelled yearly direct costs for CE in Austria were 486,598€ (95%CI 341,825€ – 631,372€). For the results of the sensitivity analysis with (a) no discounting factor for years 4–10 and (b) a discounting factor of 3% per year, see Table 4.

**Table 4:** Sensitivity analysis of overall modelled costs with 10%, 3% or no discount factor on costs per year.

**CE**	**Overall costs (95% CI)**
**Overall costs– 10% discount / year of FU**	486,598€ (341,825€ – 631,372€)
**Overall costs– 3% discount / year of FU**	515,934€ (371,931€ – 659,938€)
**Overall costs–no discount / year of FU**	531,121€ (382,196€ – 680,046€)
**AE**	
**Overall costs– 10% discount / year of FU**	683,824€ (469,161€ - 898,486€)
**Overall costs– 3% discount / year of FU**	781,077€ (548,099€ – 1,014,055€)
**Overall costs–no discount / year of FU**	838,465€ (597,826€ – 1,079,106€)

### Alveolar echinococcosis

Overall direct costs for the 7 AE patients included in this cohort amounted to 132,739€. The largest contributors to first-year costs were hospitalizations, including surgical interventions (79%), and drug therapy with albendazole (12%). Diagnostic imaging (4%), laboratory procedures (4%), and outpatient visits (<1%) contributed only marginally to the direct costs of AE in year one (see Table 5). Most costs occurred in the year of diagnosis and decreased in subsequent years (see Table 6). However, no formal statistical testing on cost differences between years was performed due to the low number of patients in follow-up in years two and three.

**Table 5:** Item-specific costs associated with the treatment of AE cases (n = 7) at an Austrian reference center for the first 3 years of follow-up. As total costs (last row) are based on a decreasing number of patients each year, this value should only be interpreted as a reference value for the proportion of costs each item is contributing.

Items	Year 1 (% of overall costs); n = 7 patients	Year 2 (% of overall costs); n = 2 patients	Year 3 (% of overall costs); n = 2 patients
**Interventions including hospitalizations**	**105,299€ (79%)**	**1,281€ (14%)**	**2,563€ (24%)**
Hepatic surgery	97,187€ (n = 4)	0€ (n = 0)	0€ (n = 0)
Other AE-related hospitalizations; diagnostic or management of complications	8,112€ (n = 4)	1,281€ (n = 1)	2,563€ (n = 2)
**Imaging**	**5,729€ (4%)**	**294€ (3%)**	**955€ (9%)**
Magnetic Resonance Imaging	1,197€ (n = 7)	171€ (n = 1)	171€ (n = 1)
Computed Tomography	1,017€ (n = 9)	113€ (n = 1)	113€ n = 1)
PET-CT	2,683€ (n = 4)	0€ (n = 0)	671€ (n = 1)
PET-MRI	832€ (n = 1)	0€ (n = 0)	0€ (n = 0)
**Outpatient visits**	**497€ (<1%) (n = 17)**	**58€ (1%) (n = 2)**	**117€ (1%) (n = 4)**
**Laboratory**	**5,007€ (4%)**	**119€ (1%)**	**516€ (5%)**
Standard laboratory packages	3,144€ (n = 12)	0€ (n = 0)	262€ (n = 2)
Serology	1,270€ (n = 30)	78€ (n = 2)	254€ (n = 6)
Histology	201€ (n = 5)	40€ (n = 1)	0€ (n = 0)
PCR	392€ (n = 4)	0€ (n = 0)	0€ (n = 0)
**Albendazole (n = packs)**	**16,207€ (12%) (n = 139)**	**7,113€ (80%) (n = 61)**	**6,646€ (62%) (n = 57)**
**Total**	**132,739€**	**8,855€**	**10,797€**

**Table 6:** Average AE-related costs in the year of diagnosis and the two subsequent years.

	**Per patient**
**AE, first year (n = 7)**	18,963€
**AE, second year (n = 2)**	4,428€
**AE, third year (n = 2)**	5,398€

Assuming 10% discounting in years 4–10, median modelled direct costs per patients with AE from diagnosis until end of follow-up after 10 years were 30,832€ (25^th^– 75^th^ percentile: 23,197€ - 31,220€) and 62,777€ (25^th^– 75^th^ percentile: 60,806€ - 67,867€) for inoperable and operable patients, respectively. Based on an estimated incidence of 12 cases per year, a mean age of 60 years at diagnosis, and a 65% probability of receiving a surgical intervention, the modelled yearly direct costs for AE in Austria were 683,824€ (95%CI 469,161€ - 898,486€). The results of the sensitivity analysis are presented in Table 4. In a sensitivity analysis with (a) no discounting factor for years 4–10 and (b) a discounting factor of 3% per year, the modelled overall costs per year were 838,465€ (95% CI 597,826€ – 1,079,106€) and 781,077€ (95% CI 548,099€ – 1,014,055€), respectively.

## Discussion

This study presents detailed direct costs associated with the diagnosis and treatment of echinococcosis in Austria, which is considered a country with very high human development by the Human Development Index. In contrast to most other economic analyses of echinococcosis, this study was based on a real cohort of patients. Nationwide costs for CE were estimated to cumulate to 486,598€ (95%CI 341,825€ – 631,372€) per year. Surgical interventions with accompanying hospitalizations and therapy with albendazole accounted for the vast majority of costs. Similar studies from high income countries are scarce. Differences in healthcare systems and billing systems, distinct methods and parameters used for the cost models of each publication, economic disparities, inflation and diverging price-levels of each country make direct comparisons almost impossible. However, the high costs of surgical interventions were also noted in a previous study from Italy [12]. Based on an Austrian population of 8.7 million inhabitants, and estimated incidence of 0.46 cases /100,000 persons / year, costs for CE accumulated to an estimated 5,660€ per 100,000 inhabitants per year (95%CI 4,006€ - 7,315€). A study from Italy estimated direct CE-associated costs of 6,398€ per 100,000 inhabitants per year with an estimated incidence between 1.06 and 2.78 cases per 100,000 inhabitants per year [13]. In one study from Spain published in 2005, the overall direct cost of CE in humans was estimated at 603,671€ (95% CI 499,200€ – 662,638€) per year [14]. Assuming a population of 43 million inhabitants, as stated in the paper, this corresponds to 1,404€ per 100,000 inhabitants per year, based on 159 diagnosed cases in 2005 (i.e. 0.36 cases per 100,000 inhabitants).

CE is a rare condition in Austria, with few endemic cases. Disability Adjusted Life Years (DALYS) lost due to CE accounted for only 0.0078% of DALYs lost to infectious and non-infection conditions in Austria as estimated by the Global Burden of Disease Study 2016 data [15]. The costs attributed to CE appear negligible when compared to major drivers of healthcare costs. For example, it was estimated that approximately 1.2 billion € are spent on healthcare costs related to cancer per year in Austria [16]. Nevertheless, morbidity and direct costs per case may be substantial, especially when considering that CE is largely preventable by appropriate livestock management, deworming of dogs, not feeding raw offal to dogs, vaccination of sheep, and good food hygiene.

As expected, costs associated with CE were significantly higher for active stages of the disease compared to inactive ones, and costs were highest in the year of diagnosis. At the center, for evaluation of interventions, advanced and relatively expensive imaging modalities, including MRI and CT scans, were performed in the management of CE to assess potential surgical operability or feasibility of percutaneous interventions [5]. Ultrasound examinations were performed, usually during an outpatient visit and were not billed separately. However, from a perspective of cost-effectiveness, replacing CT- or MRI-scans by ultrasound would only marginally reduce the direct costs of echinococcosis due to the low proportion of costs contributed by imaging in an affluent healthcare system. On the other hand, in low to middle income countries, management of CE is most probably overwhelmingly based on ultrasound, and may constitute a relevant cost-factor.

Interestingly, albendazole consumption was high for both AE and CE in the first year of follow-up when peri-interventional albendazole-therapy or first line pharmacologic therapy (depending on the cyst stage) occur. In the second and third year, however, albendazole consumption was very low in CE patients, but still high in AE patients, some of which need long term pharmacologic therapy, which is unusual in CE. Thus, overall we think that our data on albendazole consumption seem plausible and reflect treatment recommendations.

Interestingly, albendazole, a drug listed by the World Health Organization as an “essential medicine”[17], of which millions of doses are used at very low cost or through donation programs each year in developing countries, was a major driver of healthcare costs in the management of both AE and CE. Costs for albendazole are prohibitively high in some countries of Western Europe and other developed regions [18][19], and could be a primary target to decrease costs associated with the management of echinococcosis. Costs for albendazole are volatile and differ massively even between neighboring countries. In a recent study from Italy, the problem of albendazole drug shortages was also highlighted [20]. This has, to our knowledge, not yet occurred in Austria, but may potentially endanger treatment success. Multiplication of costs for old, unpatented drugs such as albendazole or daraprim following the acquisition of the main supplier by another company has possibly contributed to the high costs [21][22]. However, this is probably a problem requiring a political solution and not a medical or scientific one. Consequently, the contributors of direct costs associated with CE may be different in resource-limited regions of the world, where drug costs are lower and resources are often not available for surgical interventions.

AE is endemic in several medium to high-income countries in the Northern Hemisphere, including Austria. Median costs per patients with AE from diagnosis until the end of a 10-year follow-up period were 30,832€ (25^th^– 75^th^ percentile: 23,197€ - 31,220€) and 62,777€ (25^th^– 75^th^ percentile: 60,806€ - 67,867€) for inoperable and operable patients, respectively. This is lower than direct costs of 108,762€ per patient estimated in a Swiss study [11]. One reason for this observation is the different discount factor used (10% in our model vs. 3% in the Swiss study), which partially explains the difference. Secondly, according to the Organization for Economic Co-operation and Development (OECD), the price level index in Switzerland is 1.46 times that of Austria [23], which may account for another part of the observed difference in direct costs between these two neighboring countries. As the treatment of AE consists of major surgery or long-term albendazole therapy, with no percutaneous interventions available, cost per case was higher than for CE. This is highly plausible as CE usually does not require major surgery or long-term suppressive therapy with albendazole, which were found to be the two main drivers of costs.

Although cost per case will differ between countries, the main cost drivers for Western and Central European counties will most likely be similar, with disparities mostly attributable to difference in albendazole drug prices. This cost estimate can also be used to determine cost-effectiveness of control programs, which may include use of praziquantel-baits for *E*. *multilocularis* in foxes or systematic screening of at-risk populations [24]. Bait-based programs often involve the distribution of baits via air, and are thus expensive. Although cost estimations for such programs are not available for Austria, it seems unlikely that such interventions would be cost-effective with respect to the results of this study. Cost-effectiveness, as modelled by Hegglin et al., could only be achieved in an area with high population density after decades of campaigning [25].

There are several limitations of the current study. First, the sample size was small particularly for differentiating CE disease-stage specific costs per year in the third year of follow-up, and for AE in the second and third year of follow-up. Thus, modelled costs may be affected by individual outliers. Secondly, patient data were abstracted for a maximum of three years. Therefore, healthcare-related costs linked to echinococcosis occurring later than 3 years after diagnosis were not recorded and had to be modelled. We chose to introduce a discounting factor (10% per year) to approach this problem. This may still be relatively conservative, but is substantially higher than the previously used 3% [11]. In fact, the first year after diagnosis is the main driver of costs with later years of follow-up only contributing a relatively small proportion of costs. No deaths were observed in the study population, but excessive deaths due to echinococcosis that occurred beyond the time period based on real data (i.e. in the modelled period) were not included in the model. Excessive or earlier deaths would result in lower overall costs than modelled. Likewise, costs from patients that needed more than one intervention were included if these occurred in 2012–2014. However, relapses that may occur during the modelled period were not accounted for leading to a possible underestimation of overall costs. Measurement of albendazole-sulphoxide in blood is recommend by some guidelines for AE or in the event of adverse drug effects [5], but this was not performed during the respective period as it was unavailable at our center. Frequent measurements would slightly increase overall costs. Finally, albendazole treatment duration had to be estimated for a few cases based on current treatment guidelines [5][6], which are in line with institutional treatment guidelines.

In summary, this study presents a detailed analysis of direct healthcare-related costs linked to AE and CE in a developed country. This analysis demonstrates that hospitalizations (including surgical interventions) and albendazole treatment are the main drivers of costs for both CE and AE and, therefore, constitute potential targets for cost reduction strategies. Future studies from healthcare systems in countries with other socioeconomic backgrounds could further improve our understanding of drivers of the financial burden of echinococcosis in order to develop future and improve current prevention, treatment, and long-term care strategies.
